# The Conventions

**Published:** 1892-07

**Authors:** 


### THE CONVENTIONS.

The period of the year has nearly arrived when many dentists, throwing off the shackles of daily labor, devote a portion of their summer leisure to the advancement of their profession. The number, we regret to be forced to state, is not a large one, but every year marks a notable increase and an improvement in the character of the gatherings. This is to be expected.

The necessity for a more active interest in these meetings was never more apparent than now. Dentistry needs the help of every man connected with it, and each who has been benefited by it—and who has not?—is in duty bound to aid either in his local, State, or national organizations. It is feared that many remain away from these meetings under the impression that because they are not writers of papers or equal to taking part in discussions, they are of no use. This is a serious error. The mere presence of an individual is an inspiration, and becomes a power for good in the enthusiasm created by numbers. Too many, we fear, prefer the luxury of a rest by the sea or the mountains, under the, to us, mistaken idea that the profession will be taken care of by others. This is the selfish view of the matter, which means, generally indulged in, the relapsing of the entire body into a stage of decrepitude.

The social life of these meetings is of untold value. The yearly coming together of men from distant sections and the interchange of thought for a few days means new life in the sections they represent and an invigoration of the entire organization.

The American Dental Association meets this year at Niagara Falls, August 2. At this pleasant and convenient locality we anticipate a large gathering. At the same place, August 1, the National Board of Dental Examiners and the National Association of Dental Faculties will assemble. Matters of vital importance will claim the attention of all these bodies.

The Southern Dental Association will meet at Lookout Mountain, Chattanooga, Tenn., July 26, to which numbers from the North will be attracted, as the meetings of this organization are always full of interest.

The meeting of the American Dental Society of Europe will be held this year at Basel, Switzerland, August 1, 2, and 3, and should draw every American operator and those in sympathy with them throughout Europe to this annual gathering. Those who contemplate visiting the continent should arrange to be in Switzerland at that time. We can assure them in advance a cordial welcome. This body of earnest workers has been organized amidst many and unusual difficulties, but it has been carried along year by year until it has become a moving force in the profession everywhere.

The various State organizations will claim special interest, and will, from present indications, be well attended.

Whatever may be the changes in these annual convocations in the future, and these may not only be possible but necessary, there is but one course to take in the present,—give undivided support to those already existing.

